# Predictive value of SIRI and SII for metastases in RCC: a prospective clinical study

**DOI:** 10.1186/s12894-024-01401-2

**Published:** 2024-01-13

**Authors:** Emre Arı, Hikmet Köseoğlu, Tolga Eroğlu

**Affiliations:** Hamidiye Faculty of Medicine, Istanbul Health Practice and Research Center, Department of Urology, Health Sciences University, Istanbul, Turkey

**Keywords:** Renal cell carcinoma, SIRI, SII, Metastasis

## Abstract

**Objectives:**

In this prospective cross-sectional clinical study, we aimed to determine the efficiency of preoperative hematological markers namely SIRI (systemic inflammatory response index) and SII (systemic inflammatory index) for renal cell cancer to predict the possibility of postoperative metastases.

**Methods:**

Istanbul Education and Research Hospital, Clinic of Urology and Medical Oncology in the clinic between the dates of June 2022 to 2023 February, a diagnosis of renal cell cancer by surgical or medical oncology units imported into the treatment planning of 72 patients were included in the study. All cases with diagnoses of renal cell carcinoma were searched from hospital records. Patients with secondary malignancy, hematological or rheumatological disorders or ones with recent blood product transfusion or diagnoses of infection within the 1-month-time of diagnoses were excluded for data analyses. The data within complete blood counts (CBC) analyzed just before the time of renal biopsy or surgery were studied for SIRI and SII calculations. Twenty-two metastatic and 50 non-metastatic RCC patients were included. SIRI and SII values were compared among groups to seek change of values in case of metastasis and in non-metastatic patients a cut-off value were sought to indicate malignancy before pathological diagnosis.

**Results:**

Mean age of non-metastatic RCC patients were 60.12+/-11.55 years and metastatic RCC patients were 60.25+/-11.72. Histological sub-types of the RCC specimens were clear cell (72%), chromophobe cell (17%), papillary cell (7%) and others (4%). Median SIRI values for non-metastatic and metastatic groups were 1.26 and 2.1 (mean+/-S.D. 1.76 +/-1.9 and 3.12+/-4.22 respectively (*p* < 0.05). Median SII values for non-metastatic and metastatic groups were 566 and 1434 (mean+/-S.D. 870 +/-1019 and 1537+/-917) respectively (*p* < 0.001). AUC for detection of metastasis were 0.809 for SII and 0.737 for SIRI.

**Conclusions:**

SIRI and SII indexes seem to show a moderate efficiency to show metastases in RCC.

## Introduction

Renal cell cancer (RCC) accounts approximately 2% of global cancer diagnoses worldwide [1]. RCCs have recently been re-classified pathologically with molecular-driven criteria as well as cytoplasmic feature-based diagnoses [2]. RCC has survival from 40 to 91% according to various subtypes when non-metastatic [3]. However, these rates decreases significantly less than 20% in case of distant metastases [4].

Upon growing evidence on carcinogenesis, tumor-promoting inflammation as well as genomic instability and mutability have been suggested to be enabling characteristics of cancer [5]. Inflammatory cells have been shown to accelerate tumoral genetic evolution towards malignancy via actively mutagenic reactive oxygen species [6]. As well, inflammation have been suggested to produce molecules including growth factors, proangiogenic factors, extracellular matrix-modifying enzymes within tumoral microenvironment, thereby facilitate angiogenesis, invasion, and metastasis [7, 8].

Systemic Inflammatory Response Index (SIRI) and systemic immune-inflammation index (SII) are markers of such inflammatory tumor-supportive microenvironment. SIRI includes the counts of neutrophils, monocytes and lymphocytes with the formulation of [monocyte count x neutrophil count / lymphocyte count]. SII includes the counts of lymphocyte, neutrophil and platelet with the formulation of [ platelet count x neutrophil count / lymphocyte count] [9]. SII has been suggested to be an independent predictor of overall survival and cancer-spesific survival of patients with non-metastatic RCC [10]. Else, both SII and SIRI has been associated with advanced stages and larger tumors in localized renal cancers [11].

In this study, we aimed to evaluate predictive value of SIRI and SII for metastases in RCC.

## Materials and methods

Seventy-two patients who were diagnosed with RCC and underwent surgery in Urology Clinic and Medical Oncology Clinic of Istanbul Training and Research Hospital between July 2022 and January 2023 or were included in the treatment planning in the medical oncolgy unit were included in the study. Male and female patients older than 18 years of age who had preoperative laboratory tests and inflammatory indices could be calculated were included in the study. Information related to patients was obtained from patients’ medical records at the hospital system.

Patients were diagnosed with renal cell carcinoma through surgery or biopsy. The diagnoses of metastases of patients was determined by lymph node dissection or FDG-PET imaging.

51 of the patients were male and 21 were female. Twenty-two of the patients had metastatic RCC. 50 patients had non-metastatic RCC.

Patients older than 18 years of age, who underwent radical or partial nephrectomy due to kidney tumor and were diagnosed with RCC in their pathology, who did not undergo surgery but were diagnosed with RCC in their pathology by biopsy, and whose hematological parameter studies were performed in the last 1 week were included in this study.

Patients with another malignancy, patients who were diagnosed with or suspected infection within 1 week before admission, patients who received steroid therapy or immunosuppressive therapy at the time of admission, patients with known autoimmune disease, and patients who received blood transfusion within the last 1 month were excluded from the study.

Laboratory results and histopathological findings, tumor stages and grades of the patients of the patients included in the study were recorded. The metastatic and non-metastatic groups were compared with each other by confirming the metastasis status by imaging methods of the patients whose histopathological findings were recorded. Using the laboratory results of these two groups, inflammatory indices such as SIRI and SII were calculated and their effectiveness in terms of metastasis were compared. Statistical analyses were performed with SPSS statistics software (IBM Corp. Released 2011. IBM SPSS Statistics for Windows, Version 20.0. Armonk, NY: IBM Corp.). Comparisons of groups were done with Chi-square test and Student’s t test where appropriate. The mean values were presented with their 95% Confidence intervals. Receiver operating characteristic curve (ROC) was used to illustrate related sensitivity and specificity of ADC values. Statistical significance was set at less than 0.05. All methods were carried out in accordance with relevant guidelines and regulations. All experimental protocols were approved by university ethics committee. Informed consent was obtained from all subjects and/or their legal guardian.

## Results

A total of 72 patients who met the exclusion and inclusion criteria during the study period were included in the study as stated in the methods. Twenty (28%) of the patients were female patients and the remaining 52 (72%) were male patients. The mean age of the patients was 60.25 ± 11.72 years. The mean age for women was 62.35 ± 13.84 years, and the mean age for men was 59.44 ± 10.84 (*p* = 0.349). The mean age of metastatic patients was 60.60 ± 12.46 years, while the mean age of non-metastatic patients was 60.12 ± 11.55 (*p* > 0.05).

Twenty-two (31%) of the patients had metastatic RCC and 50 of the patients (69%) had non-metastatic RCC. The mean body mass index (BMI) of the patients was 28.10 ± 6.18. The BMI was 30.38 ± 9.27 in women and the mean BMI in men was 27.10 ± 3.93 (*p* = 0.046). While the mean BMI of the metastatic patients was 30.17 ± 10.10, it was 27.49 ± 4.41 in non-metastatic patients (*p* > 0.05).

At least 1 comorbid disease was present in 66% of the patients. According to the frequency of comorbid diseases of the patients, 44% had hypertension, 20% had diabetes mellitus, 17% had cardiovascular disease, 9% had chronic obstructive pulmonary disease and 10% had other diseases. Tumors were located unilaterally in all patients included in the study, and right and left locations (57% right and 43% left) had similar rates (*p* = 0.239).

The diagnosis was made by percutaneous renal biopsy in 8 of the patients (11%), while the diagnosis was made by surgical excision (radical nephrectomy/partial nephrectomy) in 64 cases (89%). As the surgical approach in surgical excision, laparoscopic surgery was used in 89% (57 patients) and open surgery in 11% (7 patients). Due to the fact that there were signs of metastases in the imaging performed at the time of diagnosis, a biopsy was performed on these 8 patients for verification purposes and they were diagnosed with RCC as a result of biopsy. In addition, only two patients were diagnosed with RCC by biopsy of their metastatic mass. Radical total nephrectomy was performed in 38% of patients (*n* = 24) and partial nephrectomy was performed in 62% of the remaining (*n* = 40) patients. Renal ischemia was performed in 75% of the patients who underwent partial nephrectomy, and the remaining 25% did not. Simultaneous lymph node dissection was performed in 9% (6/64) of the patients who underwent surgical excision. Surgical complication developed as pleural injury in 1.5% of the patients.

The histological subtypes of RCC specimens in our study consisted of 72% clear cell, 17% chromophobe cell, 7% papillary type and 4% other subtypes. T stages of the patients in our study consisted of 29% pT1a, 33% pT1b, 6% pT2a and 32% pT3a.

When the metastatic and non-metastatic groups were compared, statistically significant differences were observed between the two groups in terms of lymphocyte and platelet counts (*p* < 0.01) (Table 1).

**Table 1:** Comparison of patients’ blood parameter values between metastatic and non-metastatic groups

	Neutrophil count	Monocyte count	Lymphocyte count*	Leukocyte count	Thrombocyte Count*
Non-metastatic	5.73 ± 3.36	0.54 ± 0.13	2.23 ± 0.83	8.75 ± 3.64	263.19 ± 83.08
Metastatic	5.80 ± 2.04	0.65 ± 0.39	1.52 ± 0.57	8.29 ± 2.16	357.45 ± 124.47

*: *p* < 0.01

When the metastatic and non-metastatic groups were compared, statistically significant differences were observed between the two groups in terms of SIRI and SII values (*p* < 0.05 for SIRI, *p* < 0.001 for SII) (Table 2.)

**Table 2:** Comparison of SIRI and SII metastatic and non-metastatic groups from the blood parameter values of the patients

	SIRI*	SII**
Non-metastatic	1.75 ± 1.92	869.59 ± 1018.95
Metastatic	3.12 ± 4.22	1537.80 ± 916.82

*: *p* < 0.05

**: *p* < 0.001

Median SIRI values for non-metastatic and metastatic groups were 1.26 and 2.1, respectively (mean ± standard deviation 1.76 ± 1.9 and 3.12 ± 4.22 respectively (*p* < 0.05). Median SII values for non-metastatic and metastatic groups were 566 and 1434, respectively (mean ± standard deviation 870 ± 1019 and 1537 ± 917 respectively (*p* < 0.001).

**Fig. 1:**
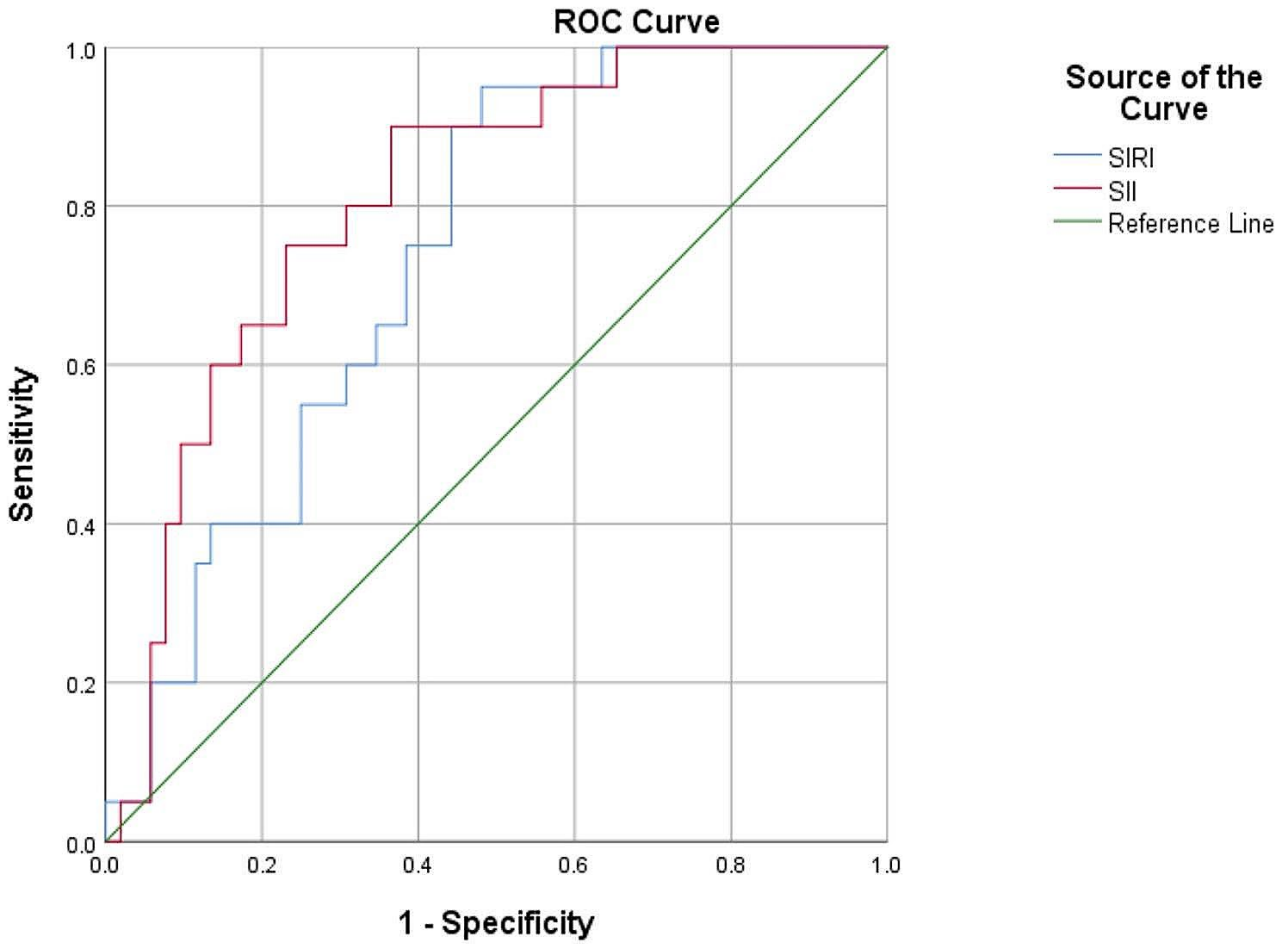
The area under the curve in metastatic patients was 0.809 for SII and 0.737 for SIRI. The ROC curve is shown in the graph

The area under the curve in metastatic patients was 0.809 for SII and 0.737 for SIRI. The ROC curve is shown in the Fig. 1. The various cut-off values, specificity, and sensitivity are shown in the Table 3.

**Table 3:** Various cut-off values, specificities, and sensitivities of SIRI and SII for indication of metastasis

	Metastasis (+) ≥ if;	Sensitivity	1 - Specificity
**SIRI**			
	1.2565	0.950	0.500
	1.2966	0.950	0.481
	1.3039	0.900	0.481
	1.3165	0.900	0.462
	1.3538	0.850	0.442
	1.3669	0.800	0.442
	1.3844	0.750	0.442
	1.4112	0.750	0.423
	1.5084	0.700	0.385
	1.5434	0.650	0.385
	1.5561	0.650	0.365
	1.6560	0.600	0.308
	1.7110	0.550	0.308
	1.7987	0.550	0.288
	2.0584	0.500	0.250
**SII**			
	504.1073	0.950	0.654
	509.4605	0.950	0.635
	516.0549	0.950	0.615
	540.6602	0.900	0.558
	546.6142	0.900	0.538
	673.8309	0.850	0.365
	701.1920	0.800	0.365
	707.2042	0.800	0.346
	744.0929	0.750	0.308
	760.6262	0.750	0.288
	949.7018	0.650	0.231
	1039.7418	0.650	0.212
	1136.2209	0.600	0.173
	1142.0997	0.600	0.154
	1200.1863	0.550	0.135

## Discussion

About one-third of patients present with metastatic RCC at the time of presentation. RCC is one of the cancers in which the immune system is most activated [12]. In order to better evaluate the outcomes of the patients, it is necessary to identify some predictive factors for reliable prognostic and metastasis prediction. In this study, thrombocyte, lymphocyte, SIRI and SII were found to be independent predictive factors in predicting metastasis from the blood parameters of the patients at the time of admission.

Increasing evidence suggests a complex interaction between leukocytes and various types of cancer, including RCC. SIRI, which is an indicator of inflammation and mainly based on peripheral neutrophil, lymphocyte and monocyte counts, was first suggested to be a reliable prognostic factor in a study conducted by Qi et al. in 2016 including 177 patients with pancreatic cancer [13].

Nebojsa et al. showed that SIRI is an independent prognostic factor for the presence of lymphovascular invasion (LVI) in a study of 491 patients who underwent cystectomy due to BC [14]. This study suggests to us that a high SIRI will contribute metastasis through LVI. Therefore this situation can also be adapted to our study.

Hu et al. conducted a study of patients with non-metastatic RCC involving 646 patients. Multivariate analysis conducted in this study has shown that SII is an independent predictor of overall survival (OS) and cancer-specific survival (CSS). In addition, it was found that SII was associated with lymphovascular invasion, positive lymph node and more aggressive phenoptype [10]. We did not compare SII of phenotypes in our study.

Zhang et al. conducted a retrospective study on 209 BC patients who underwent radical cystectomy. In this study, it was found that SII is an independent predictor for overall survival. In addition, SII was an accurate prognostic marker than neutrophil/lymphocyte ratio (NLR), platelet/lymphocyte ratio (PLR) and C-reactive protein/albumin ratio [15]. In another study, Jan et al. showed that SII was superior to NLR, PLR and monocyte-to-lymphocyte ratio (MLR) for prognostıc factor in patients with upper urinary tract cancer [16].

In the meta-analysis of patients with urological cancer, which included 14 studies with 3744 patients, it was shown that high SII value is associated with poor prognosis [9]. On the other hand, there is no study in the literature investigating the effectiveness of inflammation biomarkers in predicting metastasis in patients with RCC. In this sense, we hope that our study will contribute to the literature.

Aktepe et al., in a retrospective review of the data of 150 people with metastatic RCC who received tyrosine kinase inhibitor, showed that the PLR was superior to the NLR in terms of assessing OS [17]. In our study, when compared with the non-metastatic group, especially high platelet and low lymphocyte levels were observed in the metastatic group. In this case, it is seen that the rate of PLR is higher in the metastatic group. Therefore, high platelet count and low lymphocyte count can guide us about the risk of metastasis.

In a study by Takuya et al., in which the records of 268 nephrectomized patients were examined, it was shown that reactive thrombocytosis in renal cell carcinomas developed due to hypercytocinemia. It has also been reported that the presence of IL-6 and high CRP in the liver triggers thrombocytosis and IL-6 induces differentiation from megakaryocytes to platelets and leads to an abnormal inflammatory response. In addition, it has been stated that the tumor itself triggers thrombocytosis. It has been reported that thrombocytosis and tumor progression may also be a marker [18]. When the platelet counts were compared in our study, a statistically significant difference was observed between the metastatic and non-metastatic groups, and it is thought that it may contribute to the prediction of metastasis.

Zheng et al. investigated the relationship of SIRI with lymph node metastasis in patients with upper system urothelial carcinoma who underwent radical nephrectomy between 2003 and 2016. SIRI value was found to be associated with lymphovascular invasion and lymph node metastasis [19]. Chen et al. also investigated the association of SIRI with 3-year and 5-year survival and prognosis in clear cell RCC. They found that other inflammatory parameters, NLR, were statistically more significant than PLR values in both 3-year and 5-year follow-up [20]. In our study, we have not done any research on the comparison of SIRI, NLR and PLR values.

According to the results of the meta-analysis, which included 30 retrospective studies published between 2016 and 2020, although it was found to be associated with SIRI value, TNM stage and lymphovascular invasion, its relationship with metastasis was not evaluated. This meta-analysis study includes cohort studies of different numbers of gastrointestinal cancers, lung cancer, cervical cancer, breast cancer, urological cancer and soft tissue cancers [21]. Our study shows that SIRI can be a parameter that can be used to predict metastasis.

High SII value was found to be associated with advanced TNM stage and poor prognosis [22]. In our study, it was shown that the level of SII is associated with the risk of metastasis.

This study has several limitations. Our study involves small number of patients. More comprehensive results and possible mechanisms can be revealed by evaluating the results of more patients that can be done in this regard. First, carrying out our study with a larger group and in a wider period will contribute more to the results.

Obtaining the data of patients in a single center is one of the factors that restrict the study. A study involving multicenter patients is needed.

Some of the diagnoses of metastases established by imaging methods that is not confirmed by biopsy is one of the factor that restricted this study. The diagnoses of metastases have been performed by biopsy in 32% (7/22) and radiologically 68% (15/22). However, the diagnoses performed radiologically were clear due to properties of cross-sectional imaging including MRI and CT both of which had been proved to have high sensitivity and specificity in cases with aforementioned properties [23].

Incorporating SIRI and SII into routine assessments could provide nuanced prognostic insights, aiding clinicians in identifying patients at an elevated risk of metastatic progression. The integration of these markers may guide personalized treatment strategies, allowing for interventions tailored to an individual’s inflammatory profile. SIRI and SII could serve as valuable tools for monitoring treatment responses dynamically, offering insights into the effectiveness of specific therapeutic approaches. Combining these markers with emerging technologies, such as radiomics and genomics, may offer a more comprehensive understanding of RCC. The combination of radiomics features and genomics data has achieved good results [24]. Considering the intrinsic heterogeneity of renal lesions, the integration of both radiogenomics and hematological markers could potentially provide a more comprehensive risk stratification for RCC patients. This collaborative approach has the potential to refine predictive models for RCC metastases, improving the accuracy of prognostic assessments and guiding clinical decision-making.

As a result of our study, inflammation parameters obtained from venous blood samples taken from patients can be used to predict metastasis. Low lymphocyte, high platelet count, increased SIRI and SII values indicate a high probability of metastasis. We think that it would be beneficial to conduct more comprehensive studies based on repeated measurement results by evaluating the results of more patients.

## Conclusions

According to the results of this study, it is seen that the risk of metastasis may be higher in patients with RCC who have high SIRI and SII values, low lymphocyte count and increased platelet count, which are among the inflammatory parameters obtained from the venous blood sample at the time of diagnosis of patients with RCC. This technique is cheap and accessible.

High SIRI, SII, neutrophils and low lymphocytes at the time of diagnosis alert us in terms of metastasis research. These laboratory tests may show us the way for early recognition of metastasis in the future. We hope that laboratory tests will be able to show whether imaging is necessary for the diagnosis of metastasis of RCC in the future. A predictive model can be developed using these tests in the future. Therefore, early recognition of metastasis may be useful in planning treatment and follow up.

## Data Availability

All data is available from corresponding author on reasonable request.

## Abbreviations

AUC: Area Under the Curve
BMI: Body Mass Index
BC: Bladder Cancer
CBC: Complete Blood Count
CT: Computed Tomography
FGF-2: Fibroblast Growth Factor-2
G-CSF: Granulocyte Colony Stimulating Factor
GM-CSF: Granulocyte-Macrophage Colony Stimulating Factor
MRI: Magnetic Resonance Imaging
MSKCC: Memorial Sloan Kettering Cancer Center
NLR: Neutrophil/Lymphocyte Ratio
NF-Κb: Nuclear Factor kappa B
OS: Overall Survival
PLR: Platelet/Lymphocyte Ratio
RCC: Renal Cell Carcinoma
SII: Systemic Inflammatory Index
SIRI: Systemic Inflammatory Response Index
STAT-3: Signal Transducer and Activator of Transcription 3
TME: Tumor Microenviroment
TNF: Tumor Necrosis Factor
VEGF: Vascular Endothelial Growth Factor
